# Strategies in the synthesis of dibenzo[*b,f*]heteropines

**DOI:** 10.3762/bjoc.19.51

**Published:** 2023-05-22

**Authors:** David Irving Hermann Maier, Barend Christiaan Buurman Bezuidenhoudt, Charlene Marais

**Affiliations:** 1 Department of Chemistry, University of the Free State, PO Box 339, Bloemfontein, 9300, South Africa

**Keywords:** dibenzo[*b*,*f*]azepine, dibenzo[*b*,*f*]heteropine, dibenzo[*b*,*f*]oxepine, iminostilbene, synthesis

## Abstract

The dibenzo[*b,f*]azepine skeleton is important in the pharmaceutical industry, not only in terms of existing commercial antidepressants, anxiolytics and anticonvulsants, but also in reengineering for other applications. More recently, the potential of the dibenzo[*b,f*]azepine moiety in organic light emitting diodes and dye-sensitized solar cell dyes has been recognised, while catalysts and molecular organic frameworks with dibenzo[*b,f*]azepine derived ligands have also been reported. This review provides a brief overview of the different synthetic strategies to dibenzo[*b,f*]azepines and other dibenzo[*b,f*]heteropines.

## Introduction

**Figure 1:**
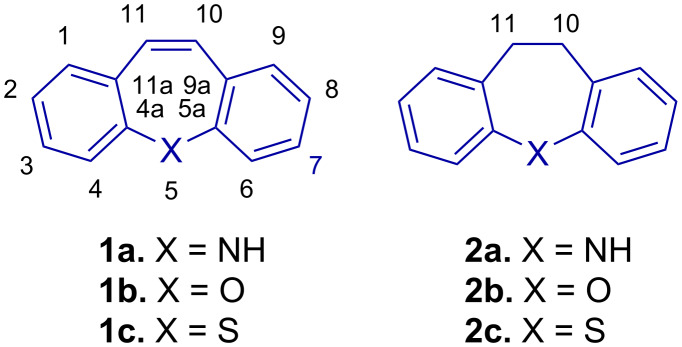
Dibenzo[*b,f*]azepine (**1a**), -oxepine (**1b**) and -thiepine (**1c**) as examples of dibenzo[*b,f*]heteropines (**1**) with the corresponding 10,11-dihydro derivatives (**2**).

The dibenzo[*b*,*f*]azepine (**1a**) scaffold (Figure 1) is featured in commercial pharmaceuticals [1] and other lead compounds [2–4], ligands [5–6] and in materials science with possible applications in organic light emitting diodes (OLEDs) [7] and dye-sensitized solar cells (DSSCs) [8–10].

**Figure 2:**
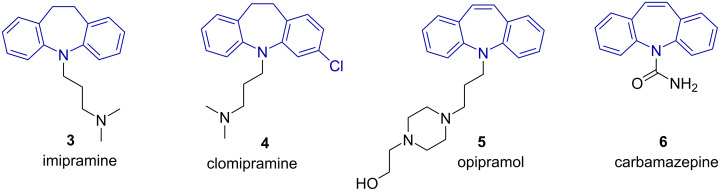
Selected pharmaceuticals with the dibenzo[*b*,*f*]azepine skeleton.

Commercial pharmaceutical agents based on dibenzo[*b*,*f*]azepine (**1a**), or the 10,11-dihydro derivative thereof (**2a**), include imipramine (**3**) and clomipramine (**4**) (tricyclic antidepressants) [11–14], opipramol (**5**) (generalized anxiety disorder) [15] and carbamazepine (**6**) (seizure disorders) [16] (Figure 2).

**Figure 3:**
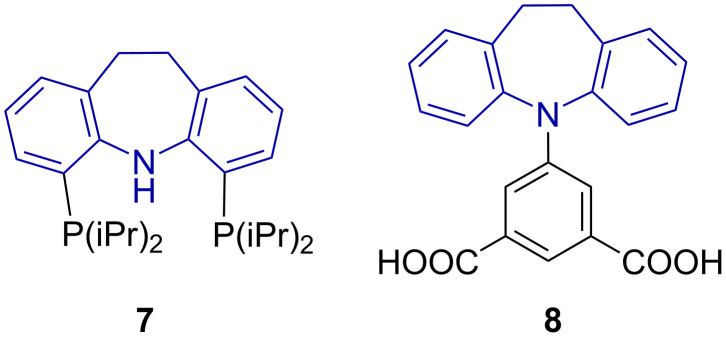
Examples of 10,11-dihydrodibenzo[*b,f*]azepine-based ligands.

10,11-Dihydrodibenzo[*b*,*f*]azepine-based ligand **7** and a methyl analogue thereof are known to form pincer complexes with Pd, Ir, Rh and Ln [5], whereas a copper(II) wagon wheel complex of **8** was reported in a molecular organic framework (MOF) (Figure 3) [6].

**Figure 4:**
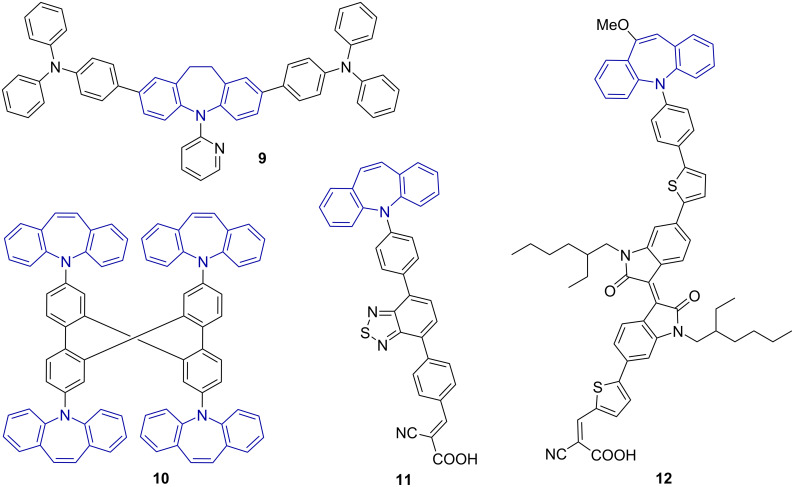
The dibenzo[*b,f*]azepine moiety in dyes with properties suitable for the use in organic light emitting diodes and dye-sensitised solar cells.

4,4'-(5-(Pyridin-2-yl)-10,11-dihydro-5*H*-dibenzo[*b,f*]azepine-2,8-diyl)bis(*N,N*-diphenylaniline) (**9**) exhibits properties suitable for the use in organic light emitting diodes [7] whereas dyes **10**–**12** were found suitable for the use in dye-sensitised solar cells (Figure 4) [8–10].

**Figure 5:**
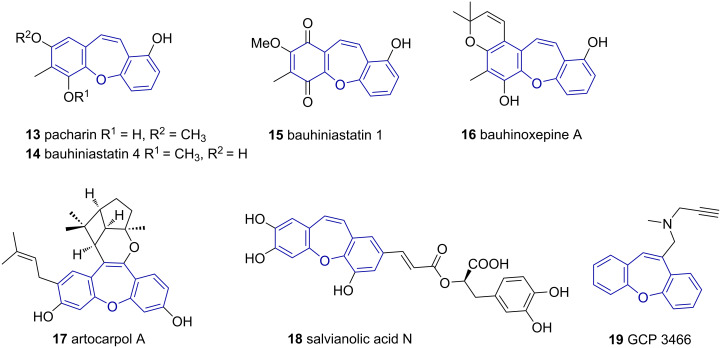
Selective bioactive natural products (**13**–**18**) containing the dibenzo[*b,f*]oxepine scaffold and Novartis CGP 3466 (**19**).

Though analogous dibenzo[*b*,*f*]oxepines **1b**, with an oxygen in the heterocyclic ring as opposed to nitrogen in azepines, are known from natural sources (compounds **13**–**18** as examples) [17–25], the application thereof in a clinical setting is limited (Figure 5). Novartis CGP 3466 (**19**), a propargylamine derivative, showed excellent neuroprotective properties for the treatment of Parkinson’s disease in rat models (Figure 5) [26]. Unfortunately, the promising preclinical studies of **19** could not be replicated in human trials [27].

For more details on the early synthesis of dibenzo[*b*,*f*]azepine (**1a**), the extensive review prepared by Kricka and Ledwith [28] in 1974, is recommended. While the review is lacking modern metal catalysis, it is still an excellent work covering early syntheses and properties. An analogous review published by Olivera et al. [29] covers the topic of dibenzo[*b*,*f]*oxepines (**1b**) up to 2002.

Other heteroatoms (e.g., O, N, S, P, B and Si) in the heterocyclic ring result in analogues of dibenzo[*b*,*f]*azepines and -oxepines. This group of compounds will thus be broadly referred to as dibenzo[*b*,*f*]heteropines (**1**).

The first section of this review will cover the synthesis of dibenzo[*b*,*f*]heteropines (**1**) and 10,11-dihydrodibenzo[*b*,*f]*heteropines (**2**). The following section will briefly touch on functionalisation of the scaffold.

While some reports are limited to the introduction of a single heteroatom, e.g., nitrogen in the case of azepines **1a** or oxygen in the case of oxepines **1b**, some approaches allow for the incorporation of a diverse scope of heteroatoms (e.g., O, N, S, P, B and Si) and may give access to a range of dibenzo[*b*,*f*]heteropines **1** using common intermediates [30–31]. Therefore, this section will be broadly organised by reaction type responsible for ring closure.

## Review

### 1 Industrial route to 5*H*-dibenzo[*b,f*]azepine (**1a**)

10,11-Dihydro-5*H*-dibenzo[*b*,*f*]azepine (**2a**), also known as iminobibenzyl (**2a**), is used as precursor for several compounds, including 5*H*-dibenzo[*b*,*f*]azepine (iminostilbene) (**1a**), and therefore will be discussed in a section on large scale industrial synthesis.

**Scheme 1:**
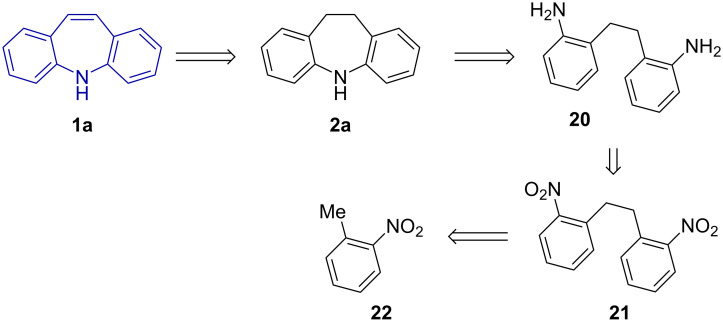
Retrosynthetic approach to 5*H*-dibenzo[*b,f*]azepine (**1a**) from nitrotoluene (**22**).

While extensive patent literature documenting methods exists, it is difficult to find accurate, up to date information regarding the industrial synthesis of 5*H*-dibenzo[*b*,*f*]azepine (**1a**) and derivatives. The following strategy (Scheme 1) was noted by chemists at Novartis as standard in 2005 [32].

- Oxidative coupling of *o*-nitrotoluene (**22**)
- Reduction to 2,2'-diaminobibenzyl (**20**)
- Ring-closing via amine condensation
- Catalytic dehydrogenation

#### 1.1 Oxidative coupling of *o*-nitrotoluene (**22**) and reduction to 2,2'-diaminobibenzyl (**20**)

**Scheme 2:**
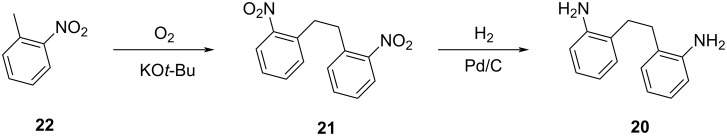
Oxidative coupling of *o*-nitrotoluene (**22**) and reduction of 2,2'-dinitrobibenzyl (**21**) to form 2,2'-diaminobibenzyl (**20**).

The preparation of dinitrobibenzyl (**21**) can be achieved by the oxidative coupling of nitrotoluene (**22**) under alkaline conditions (e.g., O_2_, KO*t*-Bu; O_2_, KOH, MeOH, ethylenediamine, etc.), as reported by Stansbury and Proops [33]. Aerobic oxidation of **22** in alkaline methanol with added ethylenediamine, gave **21** in 36% yield (Scheme 2), which is poor compared to that reported for the *p*-nitro derivative (75%). Moormann, Langbehn and Herges [34] recently optimized the method by the introduction of Br_2_ as oxidizing agent (*t*-BuOK, Br_2_, THF) to give the desired 2,2'-dinitrobibenzyl (**21**) in 95% yield.

In a method patented in 1987 [35], **22** is coupled oxidatively in the presence of a variety of transition metal (Ni, Fe, V) porphyrin catalysts and oxygen. Catalytic reduction (H_2_, Pd/C) affords 2,2'-diaminobibenzyl (**20**) in the subsequent step [28].

#### 1.2 Ring-closing via amine condensation

**Scheme 3:**
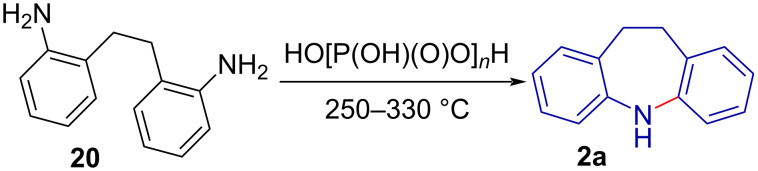
Synthesis of 10,11-dihydro-5*H*-dibenzo[*b,f*]azepine (**2a**) via amine condensation.

The initial synthesis of 10,11-dihydro-5*H*-dibenzo[*b*,*f*]azepine (**2a**) was reported in 1899 by Thiele and Holzinger [36] via the polyphosphoric acid (PPA) catalysed cyclisation of 2,2'-diaminobibenzyl (**20**) at elevated temperatures (Scheme 3) [37–38].

#### 1.3 Catalytic dehydrogenation

**Scheme 4:**
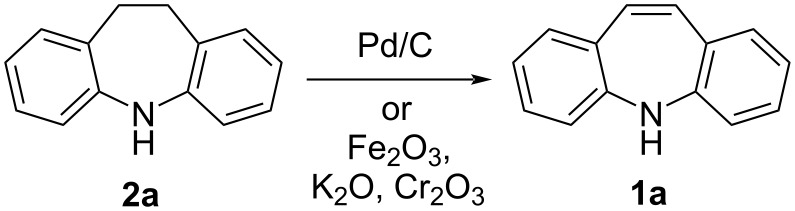
Catalytic reduction of 10,11-dihydro-5*H*-dibenzo[*b,f*]azepine (**2a**).

An early synthesis of 5*H*-dibenzo[*b*,*f*]azepine (**1a**) involved the gas phase dehydrogenation of 10,11-dihydro-5*H*-dibenzo[*b*,*f*]azepine (**2a**) to **1a** in poor yield (20–50%) [39]. The starting material **2a** was distilled through a heated (≈150 °C) column packed with Pd/C and glass wool. Crude **1a** was collected as a solid and purified. Further research has been conducted on the effect of catalyst choice and composition for large scale synthesis. Knell et al. [40–41] reported a comparison of several catalysts, which included potassium-promoted iron, cobalt and manganese oxide catalysts, for the synthesis of **1a**. Industrially, **1a** is produced by the vapour phase dehydration of **2a** over an iron/potassium/chromium catalyst system (Scheme 4) [42].

### 2 Ring expansion through rearrangement

Several methods utilise ring expansion to prepare the required 7-membered azepine and oxepine rings of **1a** and **1b**.

#### 2.1 Ring expansion of acridin-9-ylmethanols

**Scheme 5:**
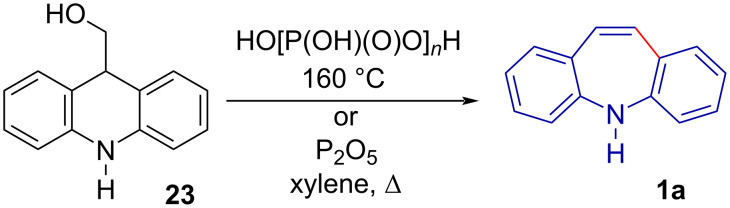
The Wagner–Meerwein rearrangement of acridin-9-ylmethanol (**23**) into 5*H*-dibenzo[*b,f*]azepine (**1a**).

In 1960, Bergmann and Rabinovitz [43] reported a simple ring expansion of acridin-9-ylmethanol (**23**) to **1a** in good yield (80%) by heating **23** in polyphosphoric acid (Scheme 5).

Independently, in an effort to synthesise phenothiazine isosteres, Craig et al. [39] prepared **1a** via a Wagner–Meerwein rearrangement of **23** with P_2_O_5_ (Scheme 5) the following year. The method was used to successfully synthesise unsubstituted as well as chloro-substituted derivatives of **1a**. Storz et al. [44] have reported on an analogous method to prepare dibenzo[*b,f*]oxepines **1b** through the rearrangement of 9-(α-hydroxyalkyl)xanthenes.

#### 2.2 Ring expansion of 2-(9-xanthenyl)malonates

**Scheme 6:**
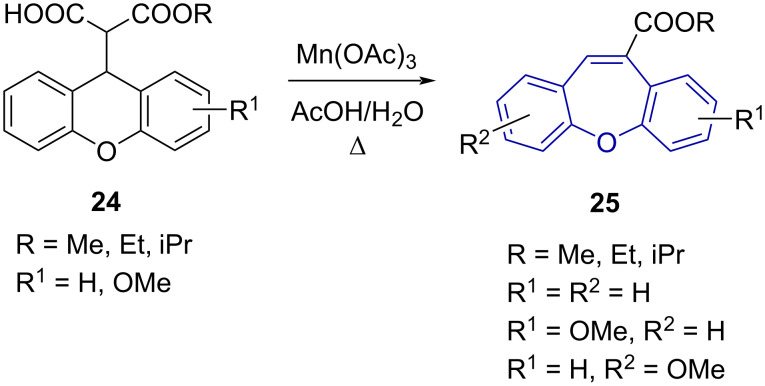
Oxidative ring expansion of 2-(9-xanthenyl)malonates **24**.

Oxidative ring expansion of 2-(9-xanthenyl)malonates **24** was reported by Cong et al. [45] as a method for the synthesis of substituted dibenzo[*b*,*f*]oxepines **25** (Scheme 6). Treatment of the malonate derivative **24** with Mn(OAc)_3_ in 90% acetic acid gave C-10 carboxylate derivatives of dibenzo[*b*,*f*]oxepine **25**. The authors proposed a one-electron oxidation of the enol carboxylate and subsequent 1,2 radical rearrangement and decarboxylation. Moderate to good yields of dibenzo[*b*,*f*]oxepine carboxylates **25** were achieved (63–85%).

**Scheme 7:**
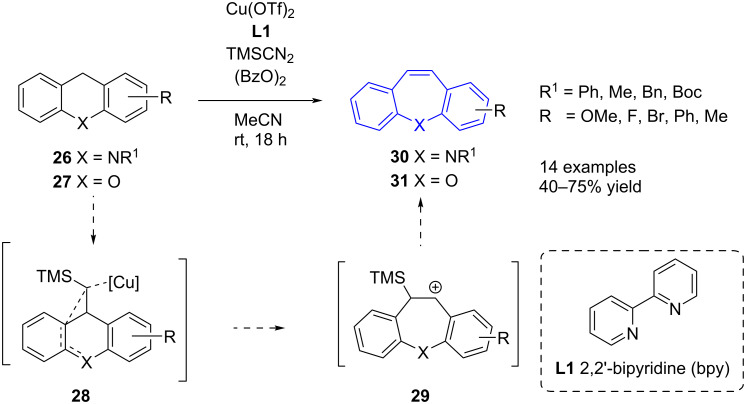
Ring expansion via C–H functionalisation.

Stopka et al. [46] reported on tandem C–H functionalisation and ring expansion as an alternative to the Wagner–Meerwin rearrangement (Scheme 7). Several azepine **30** and oxepine **31** examples were prepared in good yield from the respective acridane (**26**) and xanthene (**27**) derivatives. As an alternative to the thermal Wagner–Meerwin rearrangement (Scheme 5 and Scheme 6), which requires elevated temperatures, Stopka et al*.* [46] used mild copper-catalysed oxidative conditions to effect the transformation to **30** and **31**.

#### 2.3 Ring expansion from *N*-arylisatins

**Scheme 8:**
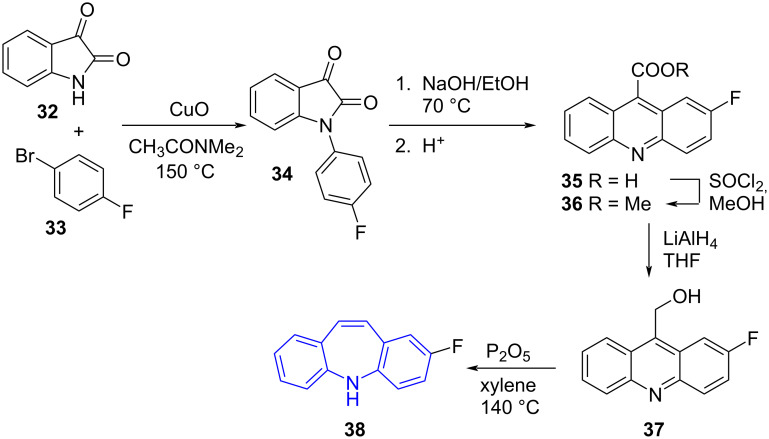
The synthesis of fluorinated 5*H*-dibenzo[*b,f*]azepine **38** from isatin (**32**).

Elliott et al. [47] reported the four-step synthesis of fluorinated 5*H*-dibenzo[*b,f*]azepine **38** from *N*-arylisatin **34** via Wagner–Meerwein rearrangement of 9-acridinemethanol **37** [43] (Scheme 8).

#### 2.4 Ring expansion of *N*-arylindoles (**41**)

**Scheme 9:**
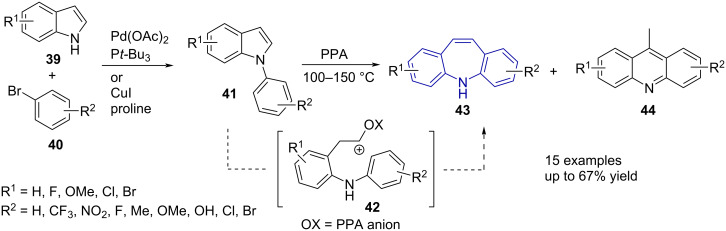
The synthesis of substituted dibenzo[*b,f*]azepines **43** from indoles **39**.

The polyphosphoric acid (PPA)-catalysed rearrangement of *N*-arylindoles **41** was first reported by Tokmakov and Grandberg [48]. The reaction provided moderate yields with a simple 2 step linear sequence from indole **39**. The reaction requires heating at elevated temperatures and reaction times of up to 150 hours. The electronic properties of the rings have a significant influence, with the strongly electron-withdrawing groups, *p*-NO_2_ and *m*-CF_3_, preventing the rearrangement and electron-donating groups (e.g., *p*-OMe, -CH_3_) promoting the rearrangement. The authors postulated an intramolecular electrophilic substitution via a carbocation intermediate **42** (Scheme 9).

Elliott et al. [47] investigated several methods to synthesise substituted dibenzo[*b,f*]azepines, which included the ring expansion of *N*-arylindoles **41** to synthesise **43** and the rearrangements of 9-acridine methanol **37** (Scheme 8) and *N*-arylindoles **41** (Scheme 9). The authors reported an excellent two-step synthesis of substituted dibenzo[*b*,*f*]azepines **43** via commercially available substituted indole **39** precursors based on the method of Tokmakov and Grandberg [48]. *N*-Arylindoles **41** were successfully synthesised via a copper-catalysed Ullman-type coupling or a palladium-catalysed Buchwald–Hartwig amination (Scheme 9). Performing the rearrangement at high temperatures resulted in the undesirable formation of acridine byproducts **44**. Cleaner reaction profiles could be obtained at a lower temperature (100 °C). In contrast to the effect reported for NO_2_ and CF_3_ substituents by Tokmakov and Grandberg [48], electron-withdrawing halogen substituents on the aryl ring did not prevent rearrangement to dibenzo[*b*,*f*]azepine **43** [49]. The isolated yield of unsubstituted **43** was good (67%), however, substitution resulted in a decreased yield. While fluoro groups were well tolerated, a major drawback of the method is the acid-catalysed dehalogenation of chloro- and bromo-substituted dibenzo[*b*,*f*]azepines. The brominated analogue was only isolated in 5% yield, compared to 67% for the unsubstituted **43**. In addition, several methods of carboxamidation were tested, thus allowing the authors to synthesize carbamazepine (CBZ) derivatives of **43**.

### 3 Metal-catalysed cyclisation

Diverse metal-catalysed coupling methods exist for the preparation of the dibenzo[*b,f*]heteropine ring system. The following approaches are broadly categorised according to the major or final catalytic step employed to form the 7-membered heterocycle as several synthetic methods use multiple catalytic steps.

#### 3.1 Buchwald–Hartwig amination, etherification and thioetherification

**Scheme 10:**
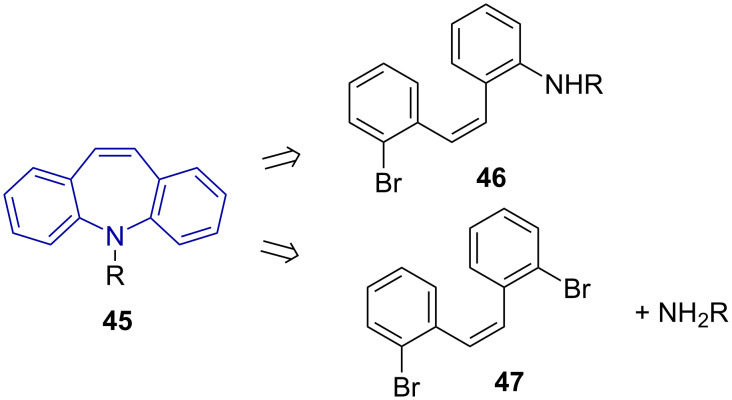
Retrosynthetic pathways to dibenzo[*b,f*]azepines via Buchwald–Hartwig amination.

The Buchwald–Hartwig reaction gives access to arylamines, -ethers and thioethers from aryl halides and triflates through palladium catalysis [50–51]. Scheme 10 provides a retrosynthesis of amination in the synthesis of dibenzo[*b*,*f*]azepine **45** as an example.

**Scheme 11:**
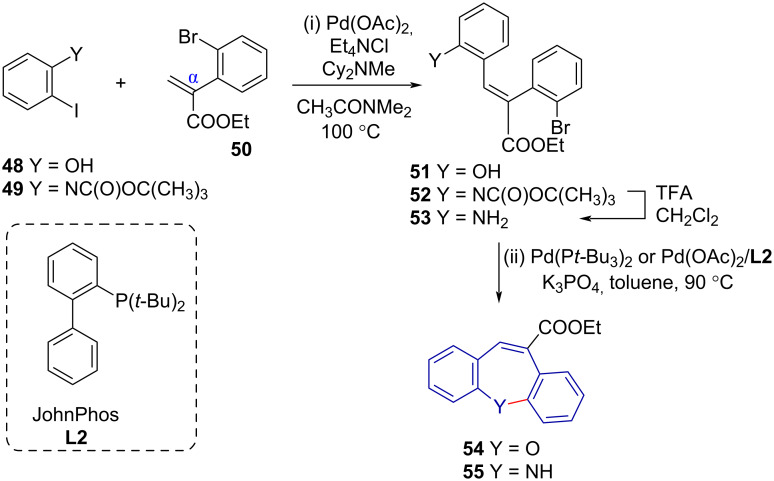
Synthesis of dibenzo[*b,f*]oxepine **54** and -azepine **55** derivatives via (i) Heck reaction and (ii) Buchwald–Hartwig-type etherification or amination.

Arnold et al. [30] reported an excellent method for the convergent synthesis of variable sized dibenzo-fused heterocycles. Among these, Heck reaction conditions allowed for the coupling of aryl acrylates **50** to aryl halides **48** and **49**, followed by intramolecular Pd-catalysed amination or etherification to give C-10 carboxylates of dibenzo[*b,f*]azepine **55** and dibenz[*b,f*]oxepine **54** in good yield (Scheme 11). However, no ring-substituted derivatives were reported. The authors used alpha-substituted acrylates to reduce the effect of poor *endo*/*exo* regioselectivity in the intramolecular Heck reaction (cf. Scheme 19).

**Scheme 12:**
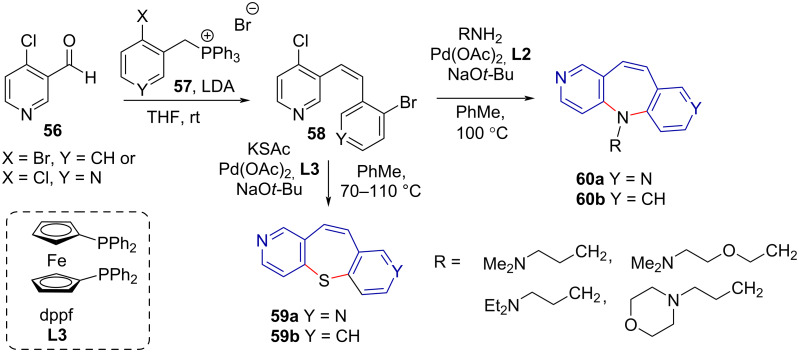
Double Buchwald–Hartwig amination and thioetherification in the synthesis of tricyclic azepines **60** and thiepines **52**.

Božinović et al. [52] reported the synthesis of symmetrical 5*H*-dipyridoazepines **60a** and unsymmetrical 5*H*-pyridobenzazepines **60b** via cyclisation of 2,2'-dihalostilbene analogue **58** through a Pd-catalysed double Buchwald–Hartwig amination. The stilbene analogues **58** were prepared by a Wittig reaction with reported yields of the desired *Z*-isomer around 55%. The amination step was performed on a series of primary alkylamines (RNH_2_) with moderate to good yields (47–87%). The strategy was also successfully applied to the synthesis of thiepines **59** with moderate yield (49–51%, Scheme 12).

**Scheme 13:**
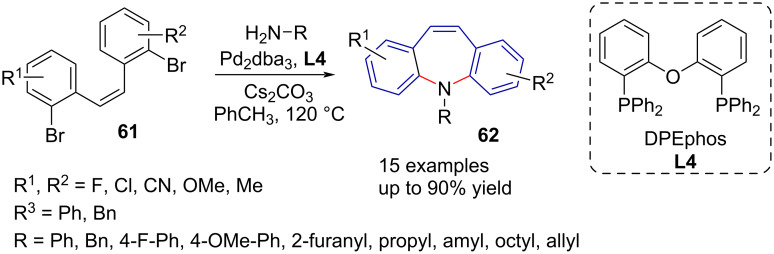
Double Buchwald–Hartwig amination towards substituted dibenzoazepines **62**.

Zhang et al. [53] applied a Buchwald–Hartwig amination in 2012 to assemble substituted dibenzo[*b,f*]azepines **62**. The reaction pathway includes the synthesis of intermediate stilbenes **61** by Wittig coupling. The authors elected to use a Pd_2_dba_3_/DPEphos (**L4**)/Cs_2_CO_3_ system (dba = dibenzylideneacetone; DPEphos = bis[(2-diphenylphosphino)phenyl] ether) in toluene after catalyst and ligand screening. Cyclisation of several substituted 2,2'-dibromostilbenes **61** by means of a double Buchwald–Hartwig amination gave yields between 62% and 96% using aniline as the amine reactant (Scheme 13). The reaction proved to be compatible with both aromatic and aliphatic amines and the reaction time varied between 11 and 24 hours. Fluoro, chloro, nitrile, alkyl, and methyl ether aromatic substituents were tolerated.

**Scheme 14:**
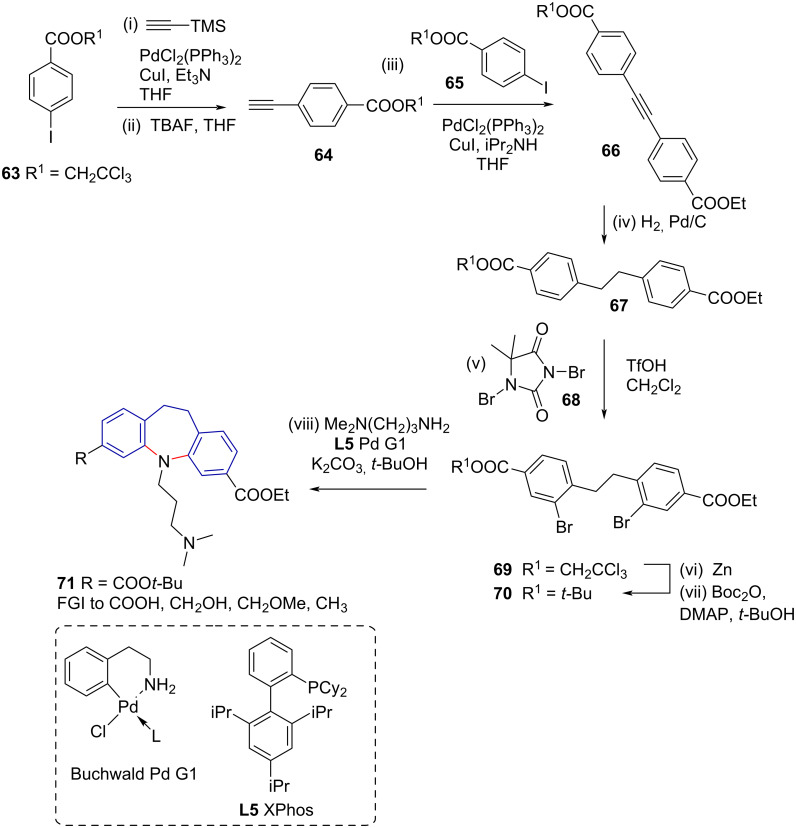
Double Buchwald–Hartwig amination towards 10,11-dihydro-5*H*-dibenzo[*b,f*]azepine derivatives **71**.

Unsymmetrical 10,11-dihydro-5*H*-dibenzo[*b*,*f*]azepine derivatives **71** have been synthesised by *ortho*-bromination of functionalised dihydrostilbenes **67**, followed by intramolecular cyclisation using Buchwald–Hartwig amination (Scheme 14) [54]. The pathway relies on a double Sonogashira coupling [(i) and (iii)], reduction (iv), and bromination (v), followed by Buchwald–Hartwig amination (viii) (Scheme 14). While interesting, the reaction has limited substrate scope due to the reliance on a late-stage bromination. To achieve the correct *ortho*-bromo substitution pattern, it requires a *para*-substituted ester as a directing group. The strategy furthermore cannot access 5*H*-dibenzo[*b*,*f*]azepines **1a** as the ethylene bridge would cross react with the brominating agent [55–56].

**Scheme 15:**
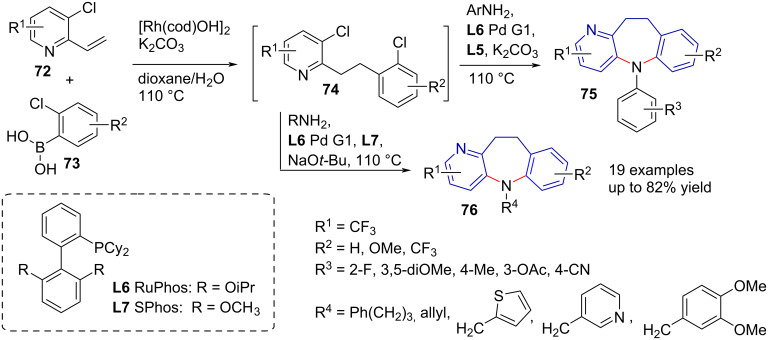
One-pot Suzuki coupling–Buchwald–Hartwig amination.

*N*-Aryl and *N*-alkyldihydropyridobenzazepines **75** and **76** were synthesised by Tsoung et al. through a multicomponent reaction system [57]. The authors provided a series of substituted derivatives through Pd/Rh-catalysed domino coupling. The reaction proceeded via a Suzuki coupling, followed by an in situ Buchwald–Hartwig amination. The authors reported moderate to good yields in a series with electron-donating and electron-withdrawing groups, as well as *N*-aryl and *N*-alkylamines (Scheme 15).

**Scheme 16:**
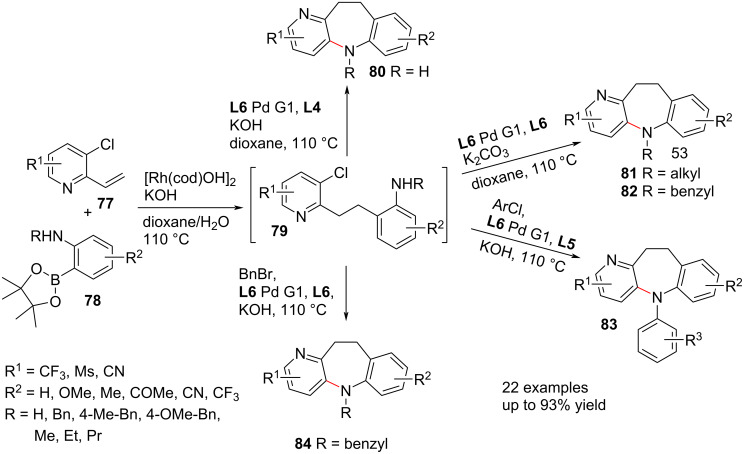
One-pot Rh/Pd-catalysed synthesis of dihydropyridobenzazepines.

Lam et al. [58] expanded on the multicomponent method to form substituted dihydropyridobenzazepines **80**–**82** wherein vinylpyridines **77** are coupled with boronate ester anilines **78** in a Suzuki reaction, whereafter Buchwald–Hartwig amination afford the various diarylazepines. A three-component one-pot process allowed for a second in situ Buchwald–Hartwig amination of the diarylazepine with aryl or benzyl halides to give the respective *N*-aryl and *N*-benzylazepine derivatives **83** and **84** (Scheme 16).

#### 3.2 Mizoroki–Heck coupling

**Scheme 17:**
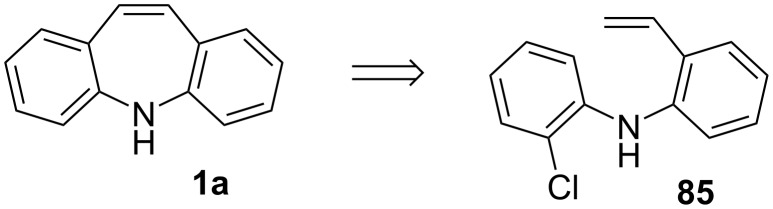
A retrosynthetic pathway to dibenzo[*b,f*]azepines via Mizoroki–Heck reaction.

Whereas Arnold et al. [30] reported the preparation of dibenzo[*b,f*]heteropines via consecutive Heck and Buchwald–Hartwig reactions (Scheme 11), amination may also precede the introduction of the double bond (Scheme 17). The formation of the dibenzo[*b*,*f*]heteropine skeleton by means of a final Mizoroki–Heck reaction will be discussed in the following section.

**Scheme 18:**
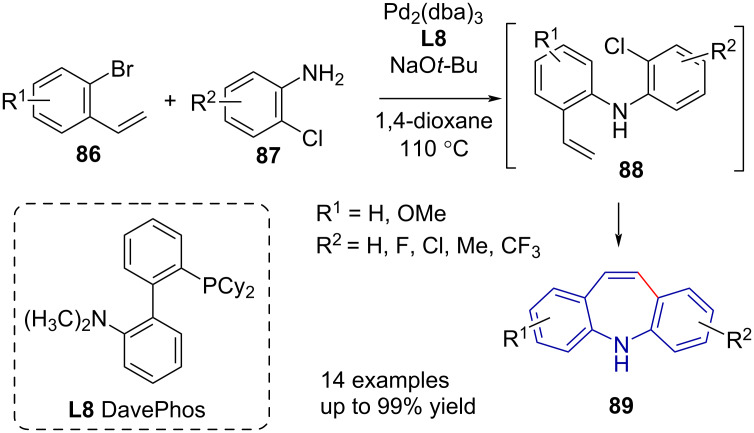
One-pot domino Pd-catalyzed Mizoroki–Heck–Buchwald–Hartwig synthesis of dibenzo[*b,f*]azepines.

The Buchwald group [59] reported a ligand-controlled divergent synthesis involving intramolecular cyclisation, allowing for the formation of several heterocycles, including dibenzo[*b*,*f*]azepines **89**, in two steps. Screening of reaction conditions during the investigation resulted in the synthesis of dibenzo[*b*,*f*]azepine **89** directly from 2-bromostyrene **86** and 2-chloroaniline **87** in up to 99% yield (Scheme 18). Several substituted dibenzo[*b*,*f*]azepines **89** and heteroaryl analogues were reported with excellent yields and regioselectivity. A later correction to the article revised the yield from 99% to 70% and with overall poorer selectivity [59]. The correction is in line with reports of poor selectivity when performing intramolecular Heck reactions (cf. Jepsen et al. [60]).

**Scheme 19:**
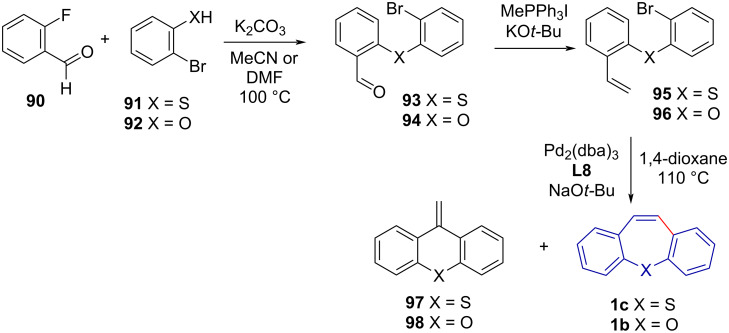
Dibenzo[*b,f*]thiapine and -oxepine synthesis via S_N_Ar (thio)etherification, Wittig methylenation and Mizoroki–Heck reaction.

An analogous reaction pathway by Jepsen et al. [60] was used to synthesise dibenzo[*b,f*]thiapines **1c** and dibenzo[*b,f*]oxepines **1b** in three steps through a styrene (**95** and **96**) intermediate (Scheme 19). While the reported conversion was excellent, the yield was low due to moderate selectivity, resulting in a mixture of 7-*endo* (**1c** and **1b**) and 6-*exo* (**97** and **98**) cyclised products.

#### 3.3 Ullmann-type coupling

**Scheme 20:**
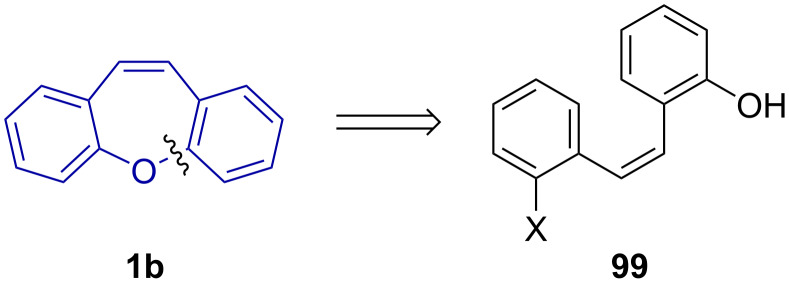
A retrosynthetic pathway to dibenzo[*b,f*]oxepines via Ullmann coupling.

Copper-catalysed Ullmann etherification (Scheme 20) offers an alternative to S_N_Ar and Buchwald–Hartwig etherification.

**Scheme 21:**
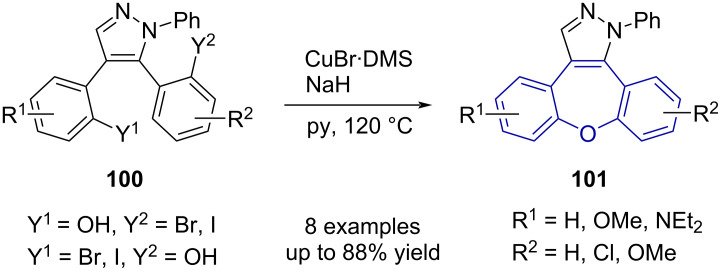
Ullmann-type coupling in dibenzo[*b,f*]oxepine synthesis.

Olivera et al. [61] reported a copper-catalysed Ullmann-type etherification as a key step in the synthesis of their pyrazole-fused dibenzo[*b*,*f*]oxepine derivatives **101** (Scheme 21).

**Scheme 22:**
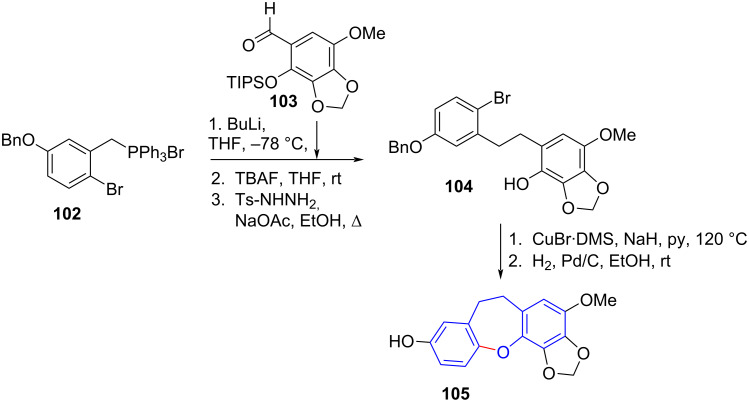
Wittig reaction and Ullmann coupling as key steps in dihydrobenz[*b,f*]oxepine synthesis.

Lin et al. [62] used copper-catalysed coupling in their total synthesis of bulbophylol-B (**105**), a substituted dihydrobenzo[*b*,*f*]oxepine. The authors synthesised an intermediate stilbene via Wittig reaction, followed by hydrogenation to give dihydrostilbene **104**, which underwent intramolecular Ullmann-type coupling catalysed by CuBr·DMS to form the fused dihydro[*b*,*f*]oxepine ring system in 89% yield, whereafter hydrogenation afforded **105** in almost quantitative yield (Scheme 22). The method is a sequence of 12 steps, the majority of which are to prepare Wittig reagent precursor **102** and the complementary aldehyde **103**.

#### 3.4 Catellani-type reaction

The Catellani reaction involves palladium-norbornene cooperative catalysis to functionalise the *ortho*- and *ipso*-positions of aryl halides by alkylation, arylation, amination, acylation, thiolation, etc. [63].

**Scheme 23:**
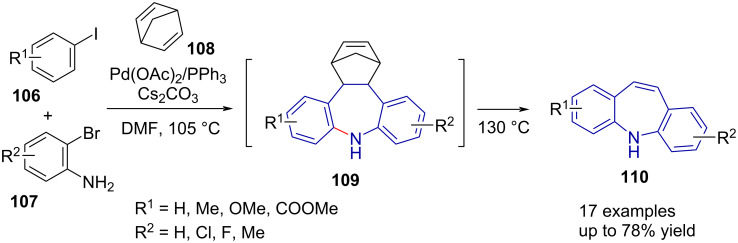
Pd-catalysed dibenzo[*b,f*]azepine synthesis via norbornene azepine intermediate **109**.

Della Ca' et al. [64] reported the synthesis of substituted dibenzo[*b,f*]azepines **110** as unexpected products during their investigation of the Catellani reaction. The Pd-catalysed reaction of an aryl iodide **106**, bromoaniline **107**, norbornadiene (**108**) and base resulted in the norbornene-azepine intermediate **109**. Heating to 130 °C induces a retro-Diels–Alder reaction, giving dibenzo[*b,f*]azepine **110** in good yield (Scheme 23). The authors synthesised a series of derivatives, with substituents including -OMe, -Me, -Cl and –F, with good yield (50–78%) in one step.

In the follow-up reported in 2018 [65], the method was extended to aryl bromides and electron-withdrawing groups. The authors found that the addition of potassium iodide, and thus in situ palladium-catalysed halogen exchange, improved the yield of dibenzo[*b,f*]azepine **110**. Unsymmetrical derivatives of **110** containing -CO_2_Me, -CF_3_, -NO_2_ and -CN substituents were synthesised in moderate to good yield (35–82%).

#### 3.5 Ring-closing metathesis

**Scheme 24:**
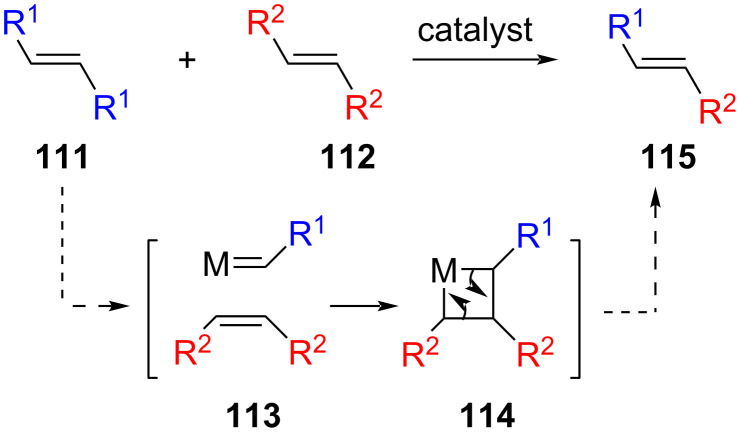
A simple representation of olefin metathesis resulting in transalkylidenation.

Olefin metathesis is a metal-catalysed reaction wherein carbon–carbon double bonds are cleaved and formed through an intermediate cyclometallacarbene **114**, thus allowing for transalkylidenation and the formation of mixed alkenes **115** (Scheme 24) [66]. Variations of this reaction include alkyne metathesis [67] and carbonyl metathesis [68].

**Scheme 25:**
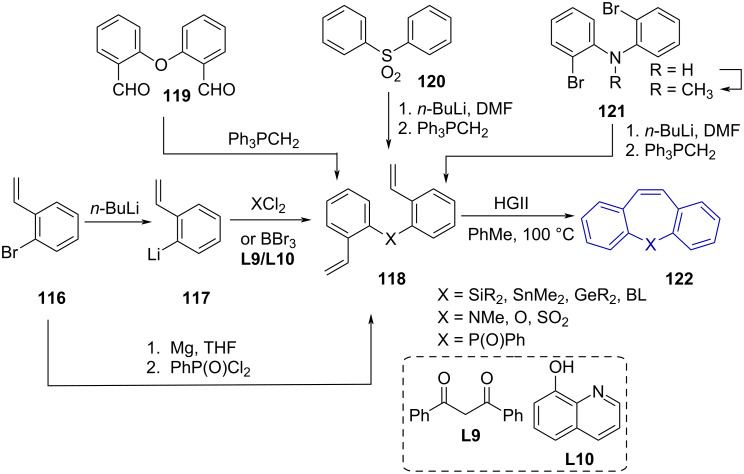
Ring-closing metathesis as key step in the synthesis of dibenzo[*b,f*]heteropines.

Ring-closing metathesis (RCM) gave access to a series of dibenzo[*b,f*]heteropines, as reported by Matsuda and Sato [31] (Scheme 25). The authors synthesised a series of Si-, Sn-, Ge- and B-tethered dienes **118** from 2-bromostyrene (**116**) via halogen–lithium exchange and quenching with the appropriate heteroatom source (SiR_2_Cl_2_, SnMe_2_Cl_2_, GeR_2_Cl_2_, BBr_3_). P-Tethered dienes were synthesised via quenching of a 2-vinylphenyl Grignard reagent with phenylphosphonic dichloride (PhPOCl_2_). O-Tethered dienes were prepared by Wittig methylenation of commercially available bis(2-formylphenyl) ether (**119**), whereas a formylation–Wittig methylenation sequence of commercial diphenylsulfone (**120**) and protected bis(2-bromophenyl)amine **121** afforded the S- and N-tethered diene, respectively. Ruthenium (2nd generation Hoveyda–Grubbs catalyst) catalysed ring-closing metathesis gave dibenzo[*b,f*]heteropines **122** in excellent yields (>80%). Unfortunately, the metathesis reaction required elevated temperatures (>100 °C) and dilute solutions to reduce unwanted self-metathesis competing with RCM. While excellent yields for synthesising the tethers and RCM products are reported, the method does not currently allow for the synthesis of unsymmetrical compounds.

#### 3.6 Alkyne–aldehyde metathesis

**Scheme 26:**
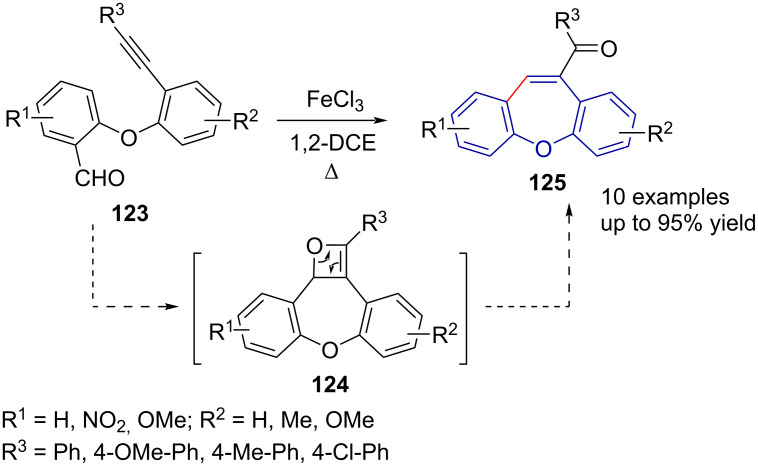
Alkyne–aldehyde metathesis in the synthesis of dibenzo[*b,f*]heteropines.

Bera et al. [69] reported on the synthesis of a series of 10-acyldibenzo[*b*,*f*]oxepines **125** by alkyne–aldehyde metathesis catalysed by iron(III) chloride (Scheme 26). Alkyne–carbonyl metathesis is proposed to proceed via [2 + 2] cycloaddition and –reversion steps, catalysed by a Brønsted or Lewis acid, with the catalyst proposed to form a σ-complex with the carbonyl group and/or a π-complex with the alkyne [68].

#### 3.7 Hydroarylation

**Scheme 27:**
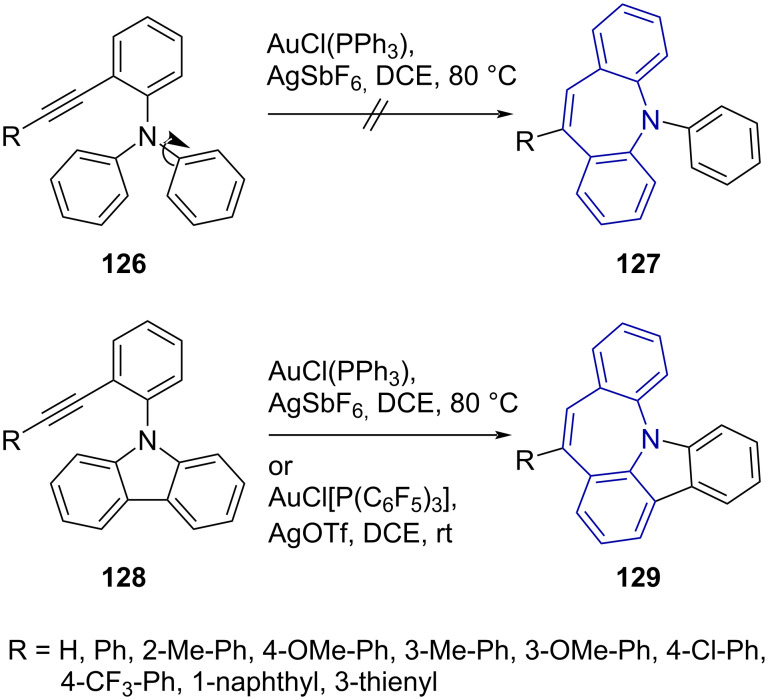
Hydroarylation of 9-(2-alkynylphenyl)-9*H*-carbazole derivatives.

The construction of an *N*-triarylated dibenzo[*b,f*]azepine scaffold **129** by means of Au(I)-catalysed hydroarylation was reported by Ito et al. [70]. While the attempted synthesis of an *N*-phenyldibenzazepine derivative **127** was unsuccessful, the authors were able to prepare a fused carbazole-dibenzo[*b,f*]azepine **129** in 90% yield via a gold/silver catalyst system (Scheme 27).

### 4 Oxidative C–C coupling

Whereas oxidative C–C coupling precedes amination in the industrial route to 5*H*-dibenzo[*b,f*]azepine, oxidative C–C coupling may also be the final step in the construction of the dibenzo[*b,f*]heteropine skeleton.

**Scheme 28:**
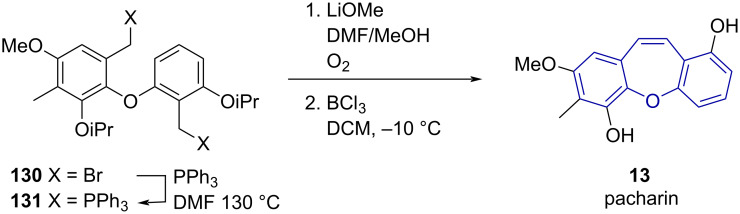
Oxidative coupling of bisphonium ylide intermediate to give pacharin (**13**).

Comber and Sargent [18] synthesised pacharin (**13**) using a novel method through oxidation of a bisphosphonium diphenyl ether prepared in situ from dibromide **130** (Scheme 28). On treatment with base and exposure to oxygen, the diylide intermediate underwent oxidative coupling to give the isopropyl-protected dibenzo[*b,f*]oxepine in good yield (65%). Subsequent deprotection of the isopropyloxy group with BCl_3_ gave **13** in good yield.

**Scheme 29:**
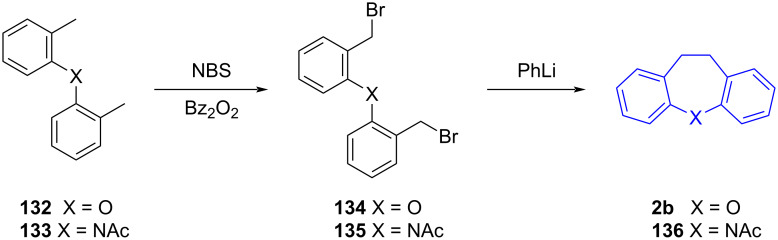
Preparation of 10,11-dihydrodibenzo[*b,f*]heteropines via intramolecular Wurtz reaction.

Bergmann et al. [71] described an early method of synthesising dihydrodibenzo[*b,f*] oxepine **2b** and -azepine **136** via a C–C intramolecular Wurtz reaction of tethered benzyl bromides **134** and **135**, prepared by benzylic bromination of the methyl substituents of **132** and **133** (Scheme 29).

### 5 1,4-Michael addition

**Scheme 30:**
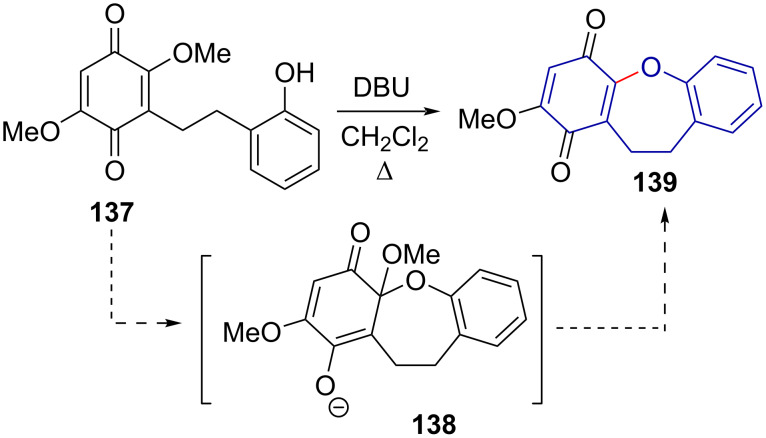
Phenol deprotonation and intramolecular etherification in the synthesis of bauhinoxepine J.

Narita et al. [72] reported their total synthesis of bauhinoxepine J (**139**), a quinone dihydrobenzoxepine derivative, by means of a base-promoted intramolecular etherification (Scheme 30).

### 6 Functionalisation of dibenzo[*b,f*]azepine

**Figure 6:**
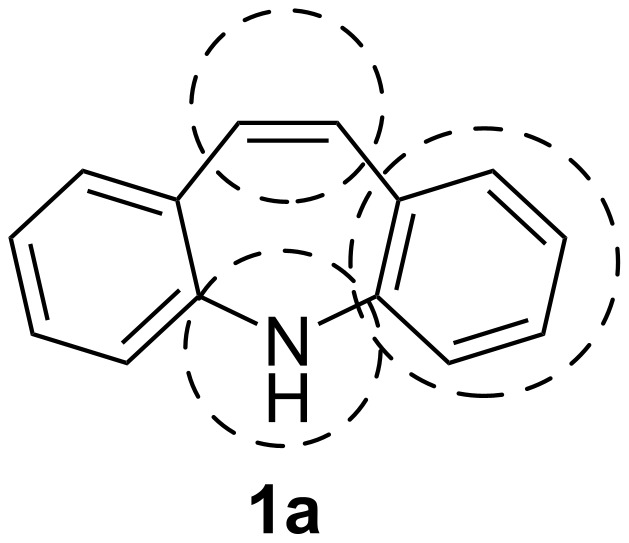
Functionalisation of dibenzo[*b,f*]azepine.

Dibenzo[*b,f*]azepine (**1a**) can be used as a precursor to complex molecules based on the dibenzazepine scaffold. Several positions of **1a** have been successfully functionalised as shown in Figure 6.

#### 6.1 N-Functionalisation

The secondary amine 5*H*-dibenzo[*b,f*]azepine (**1a**) and derivatives follow standard reactions of secondary arylamines and as such will be only briefly discussed with selected examples.

**Scheme 31:**
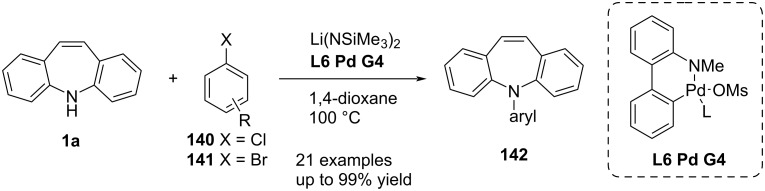
Palladium-catalysed *N*-arylation of dibenzo[*b,f*]azepine.

Huang and Buchwald [73] reported a palladium-catalysed arylation of **1a**. Treatment of **1a** with aryl halide **140** or **141** gave excellent yields of *N*-aryldibenzo[*b,f*]azepines **142** (Scheme 31). The reaction conditions were screened with several biarylphosphine ligands and Pd sources. Excellent yields were achieved with a low catalyst loading of RuPhos (**L6**) fourth generation palladacycle precatalyst **L6 Pd G4** (Scheme 31). The authors evaluated an extensive series of aryl halides. The yield proved to be good to excellent and sterically hindered aryl rings were tolerated. This method was applied by Huang et al. [74] to prepare a series of fluorescent compounds in excellent yield.

**Scheme 32:**
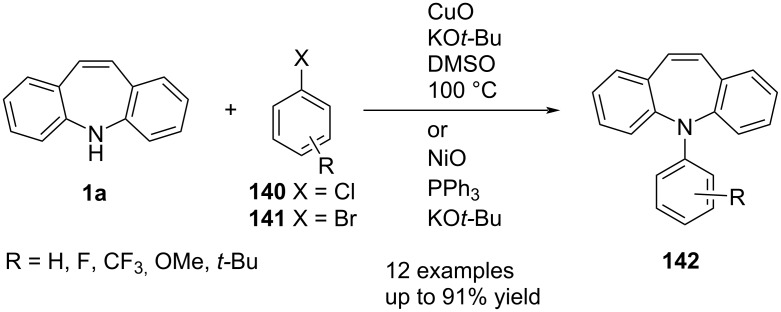
Cu- and Ni-catalysed *N*-arylation.

Copper- and nickel-catalysed arylation were reported as alternatives to the Pd-catalysed arylation of **1a** (Scheme 32). Yao et al. [75] reported the reaction of **1a** with aryl halides **140** and **141** to afford *N*-aryldibenzo[*b,f*]azepines **142** in good to excellent yields.

**Scheme 33:**
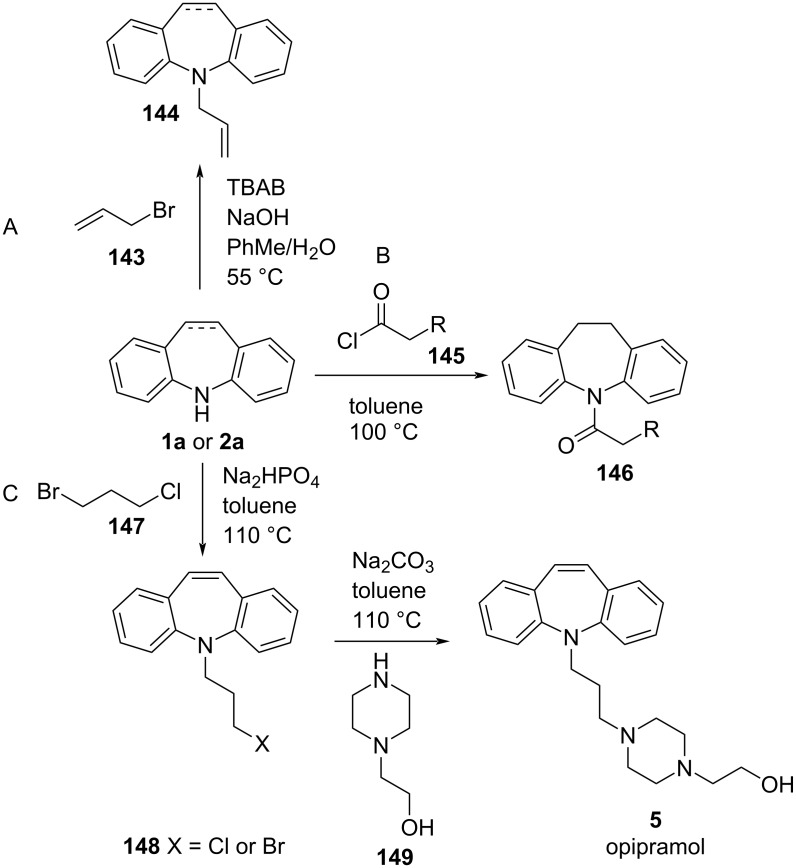
*N*-Alkylation of dibenzo[*b,f*]azepine (**1a**) and dihydrodibenzo[*b,f*]azepine (**2a**).

*N*-Alkylation of the 5*H*-dibenzo[*b,f*]azepine (**1a**) scaffold is a common point of functionalisation of **1a** and the dihydro derivative, **2a**. Indeed, the first reported synthesis of imipramine (**3**) by Schindler and Häfliger [76] proceeded by alkylation of **2a** by alkyl halides. Selected *N*-alkylations of **1a** and **2a** are included in Scheme 33.

*N*-Allylation of **1a** or **2a** with allyl bromide (**143**) can be achieved by a base-promoted substitution reaction (Scheme 33A) [77–78]. The allyl moiety in **144** allows for facile further functionalization. Amidation of the dihydrodibenzo[*b,f*]azepine (**2a**) derivatives with acyl halides **145** allowed for the introduction of variable length amide linkers by Kastrinsky et al. [3] (Scheme 33B).

An industrial synthesis of opipramol (**5**) by alkylation of **1a** was patented in 1997 [79]. The process involves the alkylation of iminostilbene (**1a**) as a critical intermediate step (Scheme 33C). The alkyl halide linker of **148** was further functionalised by reaction with piperazine derivative **149** to give opipramol (**5**).

#### 6.2 C-Functionalisation

**Scheme 34:**
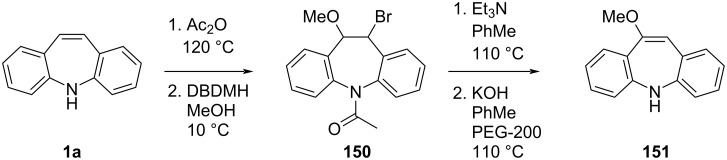
Preparation of methoxyiminosilbene.

**6.2.1 Double bond functionalisation:** Singh et al. [56] developed a large-scale synthesis of methoxyiminostilbene **151**, a precursor to the antidepressant oxcarbazepine (**153**). Bromination of acetyl-protected **1a** by 1,3-dibromo-5,5-dimethylhydantoin (DBDMH) in methanol gives the bromohydrin ether **150** in excellent yield (ca. 90%). After heating **150** with triethylamine, **151** was isolated in high yield at ca. 100 g scale (Scheme 34).

**Scheme 35:**
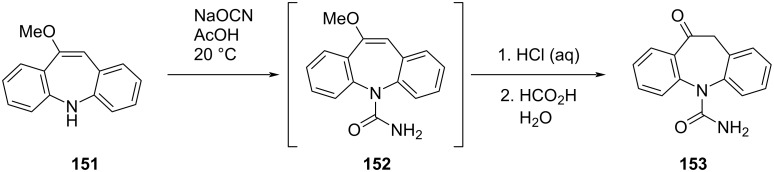
Synthesis of oxcarbazepine (**153**) from methoxy iminostilbene **151**.

The preparation of methoxyiminostilbene **151** by Singh et al. [56] complements the earlier synthesis of Fuenfschilling et al. [32] which requires **151** as an intermediate (Scheme 35). Carbamoylation of **151** gives the intermediate oxcarbazepine **152**, whereafter hydrolysis of the methyl enol ether affords oxcarbazepine (**153**) [32,56].

**Scheme 36:**
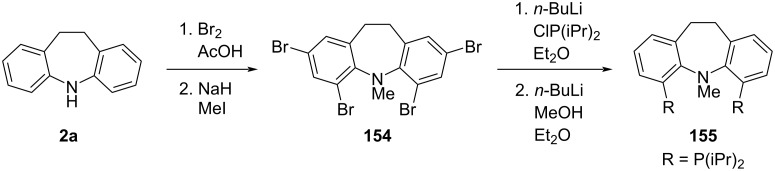
Ring functionalisation of dihydrodibenzo[*b,f*]azepine.

**6.2.2 Ring functionalisation:** Weng et al. [80] reported the synthesis of dihydrodibenzo[*b,f*]azepine (**2a**)-based pincer ligands for Rh and Ir metal complexes. The authors brominated **2a** in acetic acid, resulting in a tetrabrominated intermediate **154** in excellent yield (90%). Selective lithium–halogen exchange and reaction with a chlorophosphine, followed by debromination with BuLi/MeOH, gave the desired bisphosphine **155** in good yield (Scheme 36).

## Conclusion

The dibenzo[*b,f*]heteropine template is an important feature in several commercial and lead active pharmaceutical ingredients, biologically active natural products, dyes in OLEDs and dye sensitive solar cells, and in certain ligands. This review provides an overview of the different synthetic strategies towards dibenzo[*b,f*]azepines and other dibenzo[*b,f*]heteropines, and the functionalisation thereof. Modern metal-catalyzed methods to introduce the C–C bridge include the Heck reaction, the Sonogashira reaction, Suzuki coupling and ring-closing metathesis, whereas Buchwald–Hartwig type reactions and Ullman etherification entails the palladium or copper-catalysed formation of a carbon–heteroatom bond. Despite significant successes and facile access to the core tricyclic motif, access to dibenzo[*b,f*]heteropines with disparately substituted aromatic rings fused to the heterocyclic ring and varied substitution patterns is still limited. This void is particularly true for dibenzo[*b,f*]heteropines with multiple electron-donating substituents on both rings.
